# Cardiovascular magnetic resonance activity in the United Kingdom: a survey on behalf of the british society of cardiovascular magnetic resonance

**DOI:** 10.1186/1532-429X-13-57

**Published:** 2011-10-06

**Authors:** Renjith Antony, Marwa Daghem, Gerry P McCann, Safa Daghem, James Moon, Dudley J Pennell, Stefan Neubauer, Henry J Dargie, Colin Berry, John Payne, Mark C Petrie, Nathaniel M Hawkins

**Affiliations:** 1 Scottish National Advanced Heart Failure Service, Golden Jubilee Hospital, Agamemnon Street, Glasgow, G81 4DY, UK; 2 British Society of Cardiovascular Magnetic Resonance, BSCMR Secretariat, "Nought", The Farthings, Oxfordshire, OX13 6QD, UK; 3 University Hospitals of Leicester NHS Trust and the Leicester NIHR Cardiovascular Biomedical Research Unit, Glenfield Hospital, Groby Road, Leicester, LE3 9QP, UK; 4 National Institute of Health Research, Cardiovascular Biomedical Research Unit, Royal Brompton Hospital, Sydney Street, London, SW3 6NP, UK; 5 Institute of Cardiovascular Medicine & Science, Liverpool Heart and Chest Hospital, Thomas Drive, Liverpool, L14 3PE, UK; 6 University of Glasgow, Glasgow, G12 8QQ, UK

## Abstract

**Background:**

The indications, complexity and capabilities of cardiovascular magnetic resonance (CMR) have rapidly expanded. Whether actual service provision and training have developed in parallel is unknown.

**Methods:**

We undertook a systematic telephone and postal survey of all public hospitals on behalf of the British Society of Cardiovascular Magnetic Resonance to identify all CMR providers within the United Kingdom.

**Results:**

Of the 60 CMR centres identified, 88% responded to a detailed questionnaire. Services are led by cardiologists and radiologists in equal proportion, though the majority of current trainees are cardiologists. The mean number of CMR scans performed annually per centre increased by 44% over two years. This trend was consistent across centres of different scanning volumes. The commonest indication for CMR was assessment of heart failure and cardiomyopathy (39%), followed by coronary artery disease and congenital heart disease. There was striking geographical variation in CMR availability, numbers of scans performed, and distribution of trainees. Centres without on site scanning capability refer very few patients for CMR. Just over half of centres had a formal training programme, and few performed regular audit.

**Conclusion:**

The number of CMR scans performed in the UK has increased dramatically in just two years. Trainees are mainly located in large volume centres and enrolled in cardiology as opposed to radiology training programmes.

## Background

The indications for cardiovascular magnetic resonance (CMR) have rapidly expanded [1,2]. International and national societies provide guidance for acquisition and reporting CMR [3]. CMR training guidelines have been developed and recently updated by the Society for Cardiovascular Magnetic Resonance (SCMR) [4]. These recommendations have been adopted by the American College of Cardiology, [5] American College of Radiology, [6] European Society of Cardiology, [7] and the British Society of Cardiovascular Magnetic Resonance (BSCMR). Three levels of training are recommended by all major societies [4,5]. A recent report by the BCS working group on non-invasive imaging forecast a significant increase in the use of CMR in the following 10 years [8].

CMR is a multidisciplinary technique involving cardiologists, radiologists and radiographers. The relative contribution of each specialty to service delivery and training varies between and within individual countries. It is not known whether recommendations for acquisition, reporting and training are being applied in clinical practice. Remarkably few reports address the real life implementation of this rapidly evolving field of cardiovascular imaging. In 2010 the BSCMR initiated a national survey to examine CMR service provision and training within the UK.

## Methods

### Screening Survey

A database of all hospitals in England, Wales, Scotland and Northern Ireland was compiled from the website http://www.nhs.uk. Mental health, rehabilitation, palliative care, private and day hospitals were excluded. Every remaining hospital (n = 281) was telephoned to establish which hospitals had CMR facilities. Hospital switchboards were contacted to identify cardiology departments. The clinical director of cardiology was contacted to establish a method of contact (email, fax, or phone) to complete a 2 minute screening survey. If there was no clinical director of cardiology, a consultant cardiologist was contacted. If all cardiology contacts declined to respond, the radiology department was contacted directly to ascertain the presence of CMR capabilities. The initial screening survey identified hospitals directly providing CMR services, those with access to remote CMR services, and those without access to such services. Contact details of all CMR centres were collected for the detailed postal questionnaire.

### Detailed Survey

A detailed postal survey was sent to the lead for CMR (identified by the screening survey) at each site. The content of the questionnaire was developed with reference to the SCMR guidelines for provision of CMR services, and by consensus through the British Society of CMR including consultants specialising in both heart failure and imaging. Questions focused on establishing which specialties acquire and report CMR scans; the numbers of CMR scans performed; describing current training in the UK and assessing whether departments undertake quality control and audit.

### Response rate

Response rates were enhanced by contacting participants before sending questionnaires, employing personalised questionnaires, using hand written signatures and hand written address labels, sending by first class post, including stamped return envelopes, assuring confidentiality and using discrete identifiers on questionnaires [9,10]. Non-responders to the screening survey were followed up by telephone reminder to secretaries, with a second copy of the questionnaire offered by email or post. Secretaries were re-contacted to ensure receipt of either email or postal questionnaire. Non-response to the 2^nd ^questionnaire was followed by direct telephone calls to individual physicians and radiographers to confirm successful questionnaire delivery and intentional non-response.

### Statistical Analysis

Data were collated and analysed using Statistical Package for Social Sciences version 18 (SPSS Inc., Chicago, Illinois, USA). Data are presented as median and range for non-parametric distribution and as mean and SD with proportions expressed as a percentage of the total.

## Results

### Screening survey

Two thirds (185 of 281) of hospitals with cardiology departments responded to the screening survey. Every radiology department among the non-responders confirmed the absence of CMR facilities, validating the accuracy of screening. Nationally 60 centres with CMR were identified, one third led by cardiologists alone (33%), one third by radiologists alone (33%), and one third by both specialities (33%). All other responding cardiology departments without CMR on site referred to other centres, referring a mean of 5 patients per month. Of the 60 CMR centres identified, 53 returned the detailed questionnaire (88%). The majority of these were tertiary centres (74%). The following data apply to those 53 centres that returned a completed questionnaire.

### Equipment

The 53 responding centres have a total of 83 scanners performing CMR. The most common manufacturers were Siemens (63%), Philips (23%) and General Electric (13%). The mean scanner age was 7 years (SD 4). Of these scanners, 86% had field strength of 1.5 Tesla, 10% 3 Tesla, 1% 1 Tesla, with 3% not reported.

### Acquisition and reporting

CMR scans were usually acquired by radiographers (88%), but also by radiologists (6%), cardiologists (3%), and the remainder by trainees. Scans were reported by consultant cardiologists, radiologists, or both in 15%, 36% and 19% of centres, respectively. The remaining scans were reported by supervised trainees from cardiology, radiology or both specialties in 17%, 4% and 9% of centres respectively.

### Indications for CMR

The commonest indication for CMR was assessment of heart failure and cardiomyopathy (39%). The remainder were largely undertaken to assess coronary artery disease (predominantly viability) and congenital heart disease, accounting for 26% and 19% of scans respectively (Table 1).

**Table 1:** Service provision and indications for CMR.

Service provision	
Sessions per week (mean ± SD)	4 (4)

Patients per session (mean ± SD)	4 (3)

Number of scans per year (median ± IQR)	

• 2008	240 (100-730)

• 2009	300 (100-1000)

• 2010	332 (140-1200)

Inpatient waiting times	

• 24 hours	9 (17%)

• 48 hours	13 (25%)

• 2- 5 days	21 (40%)

• 6-10 days	6 (11%)

• > 10 days	1 (2%)

• No inpatient service	1 (2%)

• Unknown	2 (4%)

Outpatient waiting times	

• < 2 weeks	4 (8%)

• 2-4 weeks	20 (38%)

• 4-8 weeks	24 (45%)

• > 8 weeks	3 (6%)

• Unknown	2 (4%)

CMR funding	

Specific Funding	20/53 (38%)

• Primary care trust	11/20 (55%)

• Scottish Health Board	4/20 (20%)

• Tertiary	2/20 (10%)

• Shared primary care trust and tertiary	3/20 (15%)

No specific funding	33/53 (62%)

Indications for CMR (%)	

Coronary artery disease	26

• Viability	71

• Ischaemia	21

• Acute myocardial infarction	8

Heart failure and cardiomyopathy	39

Congenital heart disease	19

Acquired vascular diseases	4

Valvular heart diseases	4

Pericardial disease	4

Cardiac transplantation	< 1

Others (Tumours, chemotherapy)	4

### Service delivery and funding

**Figure 1:**
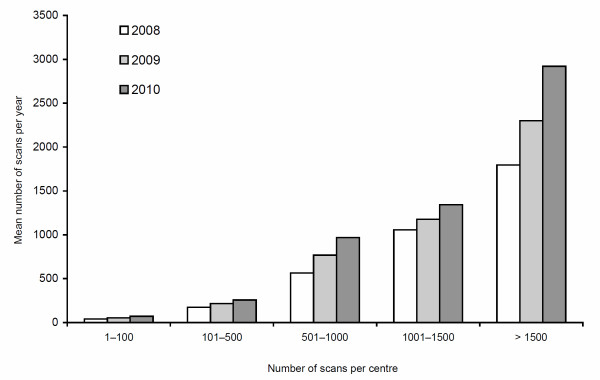
**Annual increase in mean number of scans**. Annual increase in mean number of scans performed among different volume centres.

The total annual number of scans increased from 20597 in 2008, to 31018 in 2009, to 38485 in 2010. The median number of scans performed annually in each centre rose from 240 (IQR 100-730) in 2008, to 300 (IQR 100-1000) in 2009 and again to 332 (IQR 140-1200) in 2010. The mean number likewise increased, from 557 (SD 786) in 2008, to 674 (SD 835) in 2009 and again to 802 (SD 1007) in 2010. This trend was consistent across centres of different scanning volumes (Figure 1). Respectively, in centres performing 1-100, 101-500, 501-1000, 1001-1500, and > 1500 scans annually, the mean numbers of scans per centre increased by 73%, 49%, 72%, 27%, and 63%.

**Figure 2:**
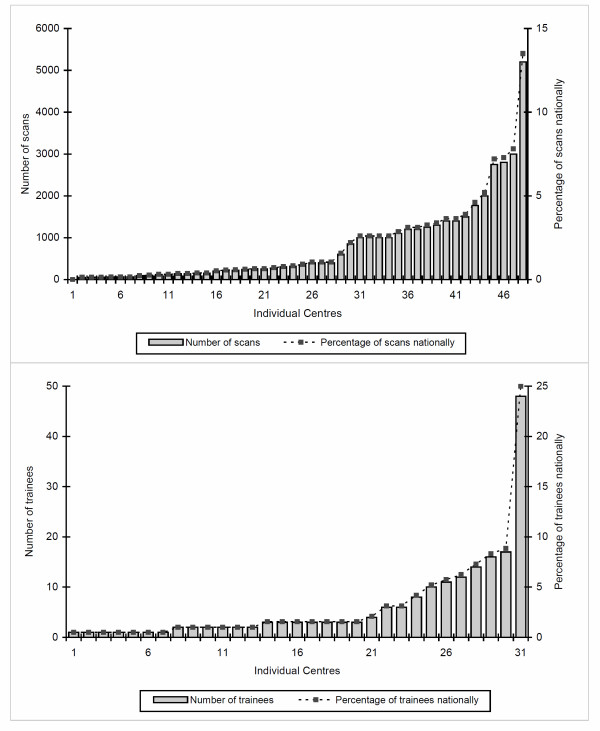
**Skewed distribution of CMR scans**. Skewed distribution of CMR scans and training per centre within the United Kingdom in 2010.

There was marked geographical variation in the number of centres with CMR and also in the number of scans performed at each of these centre (Figure 2). In 2010, twelve high volume centres (> 1000 scans annually) performed 66% of UK scans, with just six very high volume centres (> 1500 scans annually) performing 46% of scans (Figure 2). The greatest concentration of high volume CMR centres was in the South East of England. In 2010, 22 centres performed less than 300 scans.

CMR centres average 4 sessions acquiring CMR per week (a session being defined as a morning or afternoon), performing 4 (SD 3) scans per session. The median inpatient waiting time was 6-10 days and ≤ 5 days in 82% of hospitals. The median outpatient waiting time was 4-6 weeks and ≤ 8 weeks in 91% hospitals. Only 38% of centres had dedicated funding for CMR, the majority from primary care trusts or health boards in Scotland.

### Quality control and audit

Thirty four of 53 (64%) responding CMR centres undertook formal quality control; with a similar number (62%) holding a regular CMR meeting. Although regular audit (defined as an ongoing, cyclical, quality control process involving systematic review of CMR services against specified standards and implementing change if necessary) was performed in 68% of centres, this was often infrequent (Table 2). Only 14 of 50 centres performed audit on a monthly or more frequent basis. Research was conducted in nearly half of centres overall, and 100% of high volume centres (> 1000 scans annually).

**Table 2:** Research, audit and training.

Research	
Yes	26 (49%)

• dedicated CMR scanner for research	12/26 (46%)

No	26 (49%)

Unknown	1 (2%)

Audit	

Yes	36 (68%)

• Weekly	3 (6%)

• Monthly	11 (21%)

• 1-6 months	8 (15%)

• Annual	7 (13%)

• Occasional or adhoc	7 (13%)

No	16 (30%)

Unknown	1 (2%)

Training program	28/53 (53%)

• Cardiology trainees only	6/28 (21%)

• Radiology trainees only	5/28 (18%)

• Technicians only	1/28 (4%)

• Combination of above trainees	16/28 (57%)

SCMR level of training	

• Level 1 only	5/28 (18%)

• Level 2 only	6/28 (21%)

• Level 3 only	2/28 (7%)

• All 3 levels	15/28 (54%)

Level 3 Mentor	

• Yes	22/53 (42%)

• No	22/53 (42%)

• Unknown	9/53 (17%)

CMR Trainees	

Centres with trainees	31/53 (58%)

Cardiologists	166/192 (86%)

• Level 1	72/166 (43%)

• Level 2	47/166 (28%)

• Level 3	43/166 (26%)

• Unknown	4/166 (2%)

Radiologists	26/192 (14%)

• Level 1	7/26 (27%)

• Level 2	12/26 (46%)

• Level 3	3/26 (12%)

• Unknown	4/26 (15%)

Guidelines followed for trainee appraisal	

• BSCMR	4/31 (13%)

• SCMR	7/31 (23%)

• Both BSCMR and SCMR	4/31 (13%)

• Royal College of Radiologists	6/31 (19%)

• Specialist Registrar Curriculum	2/31 (6%)

• None	8/31 (26%)

BSCMR: British Society of Cardiovascular Magnetic Resonance

SCMR: Society for Cardiovascular Magnetic Resonance

### Training

Half of centres had a formal training program, the majority directed to both cardiology and radiology trainees and often accommodating trainees from both disciplines (Table 2). Overall, 32% of centres provided level 3 training and 21% level 1 or 2 training. Forty-two per cent of centres had a level 3 accredited trainer, some of which had no active trainees. A total of 192 trainees were reported at various levels of training in 31 centres, the majority (86%) being cardiology trainees. Most trainees were in England, followed by Scotland, Northern Ireland and Wales. In England, trainees were predominantly concentrated in and around London. Nationally, six centres train 61% of all UK trainees with just three centres training 42%.

Trainees were assessed against various guidelines: 23% SCMR, 13% BSCMR, 13% both SCMR and BSCMR, 19% Royal College of Radiologists, and 6% specialist registrar curriculum. Twenty six percent of centres applied no specific curriculum for trainee assessment.

## Discussion

This is the first national survey examining the provision of CMR in the United Kingdom. We have established that 60 centres provide CMR services and 53 of these (88%) responded to our detailed survey.

The number of CMR scans performed annually per centre has increased rapidly over two years, by 44% or 38% comparing mean or median numbers respectively. It is likely that demand for this imaging technique will continue to rise as cardiologists increasingly appreciate the clinical benefits of CMR. There was a marked variation in the number of scans performed in different centres. Twelve high volume centres performed 66% of all CMR scans nationally, while 28 centres in combination accounted for only 13% (Figure 2). CMR is recognised as a highly complex imaging modality and both the National Imaging Board and BSCMR/BSCI recommend a minimum number of scans per centre of 300 [11]. In 2010, 22 centres in the survey performed less than this. The survey did not address whether clinicians in these small volume centres have links to larger units. These findings certainly raise concerns regarding whether smaller volume centres have the necessary clinicians with appropriate training and volume of work to run a high quality independent CMR service.

There is a striking geographical variation in CMR use. High volume centres are concentrated in and around London with the rest of the country being populated by either low or moderate volume centres. The geographical imbalance is likely to reflect underuse outside London rather than excessive use in the capital given the large number of recognised indications for CMR. The BCS working group forecast a need to deliver 400 CMR scans per million adults by 2010, and 2275 scans per million adults by 2015 [11]. Underuse of CMR is particularly evident in centres without a scanner on site. These centres refer a mean of only 5 patients per month despite catchment areas in some cases of over 300,000.

Optimal use of CMR scanners in each centre should be ensured. Processes are required to minimise time for acquisition and intervals between patients. Clearly some protocols (for example for congenital heart disease) necessitate more time. Despite this, waiting times are generally low by UK standards, which likely reflects under-utilisation of the technique given the discrepancy between anticipated number of scans needed and the number performed. Only 15% of centres have an inpatient waiting time of greater than 5 days. Outpatient waiting lists are similarly short, with only 3 CMR centres having a waiting list in excess of 8 weeks. It is likely that rapidly increasing numbers of CMR scans nationally will be reflected in increased waiting times in the coming years unless adequate forward planning is put in place. Only 38% of services are specifically funded for CMR, the most common source being primary care trusts. Changes to commissioning systems in the National Health Service will make CMR funding a contentious issue in forthcoming years.

Cardiologists and radiologists operate CMR in isolation in 33% and 33% of hospitals respectively and collaborate to run services in 33%. Radiologists alone report twice as many scans as cardiologists alone (36% v 15%). The BSCMR advocate a joint speciality approach, as both disciplines bring complementary knowledge to CMR. Cardiologists are trained in all aspects of cardiac diagnosis and treatment, including other cardiac imaging modalities. Radiologists have formal training in extra-cardiac imaging as well as the heart. The finding that 86% of trainees are cardiologists and 14% are radiologists reflects current training patterns in CMR. Initiatives to engage radiology trainees in CMR are required to address this imbalance.

Our data suggest that 30% of centres rely on trainees to report CMR scans. We do not have information on the level of accreditation (if any) that these trainees have, although it is likely that some will have SCMR Level 2 or 3 accreditation. Current guidelines suggest that CMR scans be reported by SCMR Level 2 or 3 accredited practitioners, with no stipulation that the practitioner should be a consultant (the lead doctor in charge of patient care in the UK). This seems reasonable given that imaging modalities such as echocardiography are reported by technicians and trainees, typically supervised by a consultant cardiologist who provides advice with difficult cases and quality control. A similar arrangement was observed in all responding centres, with trainees always reporting CMR scans under consultant supervision, and consultants countersigning final reports.

The most common indications for CMR are heart failure and cardiomyopathy, coronary artery disease, and congenital heart disease. As all cardiac departments manage these conditions, it is necessary for clear referral pathways for recognised indications between district general (regional community hospital) cardiologists and regional CMR services.

Only 64% of centres have quality control processes in place, while audit is infrequent when performed at all. We regard quality control and audit as an essential component of any service. A departmental meeting should also be a feature of all CMR centres, especially for those with a commitment to training [4]. While 58% of centres have trainees, only 53% have a formal training programme, and only 42% have a level 3 mentor. We propose that centres that train should be high volume (the BSCMR stipulates 500 scans per year), [11] be supervised by a level 3 Mentor, have a formal training programme and perform regular quality control and audit. The geographical variation in CMR volume is also seen in the numbers of trainees. Sixty-one per cent of trainees are trained in only 6 centres. Indeed, three of these centres train 42% of all those trained nationally.

A number of limitations merit consideration. Our findings only capture CMR activity from 88% of UK CMR centres. Private hospitals were not included in the current survey. Our primary contact in each centre was a cardiologist or a clinical director in cardiology. Although this potentially introduces bias, we expect clinical directors or cardiologists would be aware of the presence of CMR within their institution. There is no national collection of CMR scanning figures and we have no means to determine whether the figures returned are accurate. No data was collected regarding stress imaging, or the specific number of scans performed for research as opposed to clinical indications. The provision of clinical CMR is therefore likely to be underestimated given the high academic output of several centres in the UK. The current survey informs us about UK CMR practice only. It is likely that diverse patterns would be found in other countries. Some are likely to be similar to the UK (e.g. Germany) but provision in others markedly at variance. We propose that national and international surveys should be performed using the same methodology as in the current manuscript. A trade-off exists between questionnaire length and response rate, with more questions risking increasing non-response, loss of precision and possible bias [12,13]. Only questions that were necessary to achieve the study objectives were included.

## Conclusions

CMR is a rapidly expanding imaging modality in the UK. The numbers of CMR scans are not evenly spread throughout the country but are concentrated in high volume centres. Most CMR training is provided by these centres. Centres without CMR on site refer very few patients for CMR. This has generated inequality in both service and training in the UK. The cardiology community and commissioners should therefore strive to provide a quality, equitable CMR service throughout the UK. Further work is required by the imaging societies to ensure that high quality services are delivered during the growth of this rapidly maturing modality. Future UK surveys are needed to monitor growth in CMR and surveys from other countries would be valuable as a comparator.

## Competing interests

The authors declare that they have no competing interests.

## Authors' contributions

RA designed questionnaires, collected data, analysed and interpreted the results, drafted the manuscript. MD and SD collected data and drafted the manuscript. MCP conceived the project and designed the study, drafted the manuscript, provided important intellectual content and supervised the survey. NMH designed questionnaires and survey, drafted the manuscript, provided important intellectual content and supervised the survey. GPMc designed questionnaire, provided intellectual content, drafted manuscript. JP designed questionnaire, drafted manuscript, provided intellectual content. DJP, HJD, CB, SN and JM helped design questionnaire and drafting of manuscript. All authors read and approved the final manuscript.
